# Associations between cardiovascular risk factors, disease activity and cardiorespiratory fitness in patients with inflammatory joint disease: a cross-sectional analysis

**DOI:** 10.1186/s13102-023-00678-4

**Published:** 2023-04-21

**Authors:** Kristine Røren Nordén, Anne Grete Semb, Hanne Dagfinrud, Jonny Hisdal, Sigrid Ødegård, Joseph Sexton, Camilla Fongen, Jon Skandsen, Thalita Blanck, George S. Metsios, Anne Therese Tveter

**Affiliations:** 1 Center for Treatment of Rheumatic and Musculoskeletal Diseases (REMEDY), Diakonhjemmet Hospital, Postboks 23, 0319 Vinderen, Oslo, Norway; 2 Institute of Health and Society, Faculty of Medicine, University of Oslo, Oslo, Norway; 3 Norwegian National Advisory Unit on Rehabilitation in Rheumatology, Division of Rheumatology and Research, Diakonhjemmet Hospital, Oslo, Norway; 4 The Preventive Cardio-Rheuma Clinic, Center for Treatment of Rheumatic and Musculoskeletal Diseases (REMEDY), Diakonhjemmet Hospital, Oslo, Norway; 5 Department of Vascular Surgery, Oslo University Hospital-Aker, Oslo, Norway; 6 Institute of Clinical Medicine, Faculty of Medicine, University of Oslo, Oslo, Norway; 7 Norwegian National Unit for Rehabilitation for Rheumatic Patients with Special Needs, Division of Rheumatology and Research, Diakonhjemmet Hospital, Oslo, Norway; 8 Patient Advisory Board, Center for Treatment of Rheumatic and Musculoskeletal Diseases (REMEDY), Diakonhjemmet Hospital, Oslo, Norway; 9 Department of Nutrition and Dietetics, University of Thessaly, Trikala, Thessaly, Greece

**Keywords:** (MESH terms) Cardiorespiratory fitness, Cardiovascular diseases, Rheumatoid arthritis, Spondyloarthritis

## Abstract

**Background:**

Inflammatory joint diseases (IJD) are accompanied by an increased risk of cardiovascular disease (CVD). Cardiorespiratory fitness (CRF) is a modifiable CVD risk factor and low levels of CRF associate with an elevated CVD risk. This study aimed to investigate the associations between CVD risk factors, disease activity and CRF in patients with IJD and to explore differences between patients with normal versus low levels of CRF.

**Methods:**

CRF was measured as peak oxygen uptake (VO_2peak_) with a cardiopulmonary exercise test. Participants were also evaluated for: Body composition, blood pressure, blood lipids, inflammatory markers and disease activity. Patient-reported use of cigarettes/snuff, medication, disease duration, pain, fatigue, CVD history, habitual physical activity and exercise beliefs and self-efficacy were collected by questionnaire. Cross-sectional associations between CVD risk factors, disease-related factors and CRF were analyzed by multiple linear regression. CRF was categorized to normal CRF (VO_2peak_ ≥ 80%) or low CRF (VO_2peak_ < 80%) according to age- and gender-stratified reference data. Differences in demographic, CVD and disease-related factors between patients with normal versus low CRF were explored.

**Results:**

In 60 Norwegian patients with IJD [34 females, age 59 years (IQR: 52–63)], mean VO_2peak_ was 30.2 (± 6.9) mL/kg/min, corresponding to 83% (± 18) of normative reference values. Age (coefficient: − 0.18 years, *p* = 0.01) and fat mass (coefficient: − 0.67 %, *p* < 0.001) were inversely associated with CRF, while physical activity index (coefficient: 0.13 points, *p* = 0.05) was positively associated with CRF (R^2^ = 0.66). There were no significant associations between CRF, classical CVD risk factors and disease-related variables. Compared to patients with low CRF (*n* = 30), patients with normal CRF (*n* = 30) had higher peak oxygen uptake (+ 9.4 mL/kg/min, *p* < 0.001), high-density lipoprotein cholesterol (+ 0.5 mmol L^−1^, *p* < 0.001), and exercise self-efficacy (+ 6.9, *p* < 0.01) as well as lower fat mass (− 8.7%, *p* < 0.001), resting heart rate (− 8.0 beats/min, *p* < 0.01) and triglycerides (− 0.5 mmol L^−1^, *p* < 0.01).

**Conclusions:**

In this sample of IJD-patients, age, fatmass and physical activity level were associated with CRF. CRF was lower than reference values and patients with normal CRF presented with a more favorable health profile. There is a continued need for exercise interventions to improve CRF in patients with IJD.

*Trial registration:* NCT04922840.

## Background

Inflammatory joint diseases (IJD), including rheumatoid arthritis (RA), spondyloarthritis (SpA) and psoriatic arthritis (PsA) are non-communicable diseases that present with a wide range of clinical symptoms, including joint inflammation, pain, fatigue and functional impairments. Furthermore, patients with IJD have an increased risk of cardiovascular disease (CVD), in part due to a high prevalence of traditional CVD risk factors such as hypertension, dyslipidemia and obesity [1]. Systemic inflammation driven by immunological pathways can accelerate atherosclerotic CVD, and is recognized as an independent CVD risk magnifier in the context of IJD [2, 3].

Cardiorespiratory fitness (CRF) is identified as a modifiable prognostic factor for CVD-morbidity, but common CVD risk algorithms do not include CRF in the risk stratification [4]. Routine measures of CRF as a clinical vital sign has been recommended, but seldom realized in patient clinical care [5]. Accordingly, clinicians use traditional variables such as smoking habit, blood pressure, lipid profile, age, sex and anthropometry to stratify CVD risk in patients with IJD [2]. Efforts to improve CVD risk prediction by including rheumatic disease characteristics have so far proven inadequate in accounting for the elevated CVD risk profile driven by IJD [6]. However, consistent testimony of CRF as a strong health predictor dictates a need for increased awareness on the role of CRF in overall health for patients with IJD.

Extensive epidemiological data on CRF as an independent CVD risk modifier [7–9] underlie public health recommendations for all adults to participate in regular physical activity and exercise to enhance CRF and mitigate CVD risk [10–12]. Notably, patients with IJD tend to be less physically active and present with inferior levels of CRF compared to healthy peers [13–16]. Patients with increased disease activity may gravitate towards a sedentary lifestyle and, over time, physical inactivity can precipitate a decline in CRF [17–20]. The relationship between disease activity and CRF has not been extensively investigated, and given that both low levels of CRF and an elevated inflammatory burden may increase CVD risk in IJD, the potential association between disease activity and CRF warrants further investigation.

Emphasis on the elevated risk of CVD in IJD has led to a considerable amount of reports on traditional CVD risk factors in this patient population [21–25]. Despite a strong and inverse correlation between CRF levels and CVD risk in the general population, there is a lack of evidence regarding associates of CRF in IJD and only a handful of studies have examined CVD profile and disease activity in concert with CRF in this patient population [15, 26–30]. Aforementioned studies have employed various measures of CRF and there is a need to replicate study results using the CRF criterion method. Uncovering associates of CRF in IJD may aid healthcare practitioners in identifying patients that can benefit from a CRF assessment to provide a more comprehensive assessment of CVD risk.

The primary aim of this paper was, therefore, to investigate factors that may associate with CRF in a contemporary IJD population. We hypothesized that we would uncover associations between classical CVD risk factors, disease activity and CRF in patients with IJD.

The second objective was to evaluate CRF in patients with IJD relative to reference data from the general population, and explore potential differences in demographic, cardiovascular and IJD-related factors in patients with normal versus low levels of CRF.

## Methods

### Study design and participants

The data underlying this paper stems from baseline visits in the ExeHeart trial (ClinicalTrials.gov NCT04922840); a randomized controlled trial with a primary aim to evaluate the effect of high-intensity interval training on CRF in patients with IJD [31]. Ethical approval of the ExeHeart trial, including the present study, was obtained from the Regional Committee for Medical and Health Research Ethics (201227) and the Data Protection Officer at Diakonhjemmet Hospital (reg.no. 00397). Patients willing to participate signed an informed consent form before enrolling in the study. All procedures conformed to the Helsinki declaration.

Patients were recruited from the Preventive Cardio-Rheuma Clinic, Center for treatment of Rheumatic and Musculoskeletal Diseases, Diakonhjemmet Hospital, Norway. Patients presenting with an IJD diagnosed by rheumatologist, age 18–70 years, body mass index 18.5–40, ability to walk unaided for ≥ 15 min and Norwegian or English fluency were eligible for inclusion. Exclusion criteria were sustained lower extremity injury or surgery in the past 12 months, primary neurological disease, cognitive disability, presence of one or more contraindications to maximal exercise testing as defined by the American College of Sports Medicine [10] and prior participation in high-intensity interval training ≥ 1/week in the past 3 months. Baseline study visits were carried out from August 2021 to August 2022.

### Procedures

Data collection procedures are fully detailed in the ExeHeart trial protocol [31]. In short, study variables were assessed as follows:

Medical background information including IJD diagnosis and co-morbidities were collected from the patient’s medical record. CRF was assessed by a cardiopulmonary exercise test (CPET) on a treadmill. A modified Balke [32] continuous ramp protocol was applied with initial speed individualized to the patient’s preference. Breath-by-breath gas analysis and 12-lead electrocardiography were measured continuously throughout the exercise test. Blood pressure was recorded every second minute and blood lactate was drawn from the fingertip within one minute of test completion. CRF was quantified as peak oxygen uptake (VO_2peak_), defined as the highest 30-s average VO_2_ at any point during the exercise test. In the absence of a VO2 plateau as primary end criteria, Borg rating of perceived exertion 0–10, respiratory exchange ratio, percent of age-predicted peak heart rate (220-age) and post-exercise capillary blood lactate were used to assess level of maximal effort [33]. Normative CPET data nuanced to age and gender facilitated the clinical interpretation of the exercise tests [34], and national reference data was applied to reflect our sample under scrutiny [35].

Body weight, total fat mass, total fat free mass and visceral fat indicator were quantified by bioelectrical impedance analysis. Height was registered by stadiometer, and body mass index (BMI) was calculated as body weight in kilograms divided by the square of height in meters (kg/m^2^).

Resting heart rate, systolic- and diastolic blood pressure were measured in a supine position by an ambulatory blood pressure monitor and defined as the mean of two single measurements interspaced by 30 s. Non-fasting blood samples were analyzed for total cholesterol, high-density lipoprotein cholesterol (HDL-c), low-density lipoprotein cholesterol (LDL-c), triglycerides, erythrocyte sedimentation rate (ESR) and C-reactive protein (CRP).

Clinical measures of disease activity were assessed by Disease Activity Score-28, Disease Activity Index for Psoriatic Arthritis or Ankylosing Spondylitis Disease Activity Score for patients with RA, PsA and SpA, respectively. Disease activity was further categorized to remission, low, moderate and high as previously described [31].

Patients answered a digital questionnaire accommodated by the University of Oslo (nettskjema@ usit.uio.no). In the present study, we included questionnaire items addressing personal background information, current use of cigarettes and/or snuff, IJD disease duration, pain and fatigue (Numerical Rating Scales 0–10), CVD history and use of medication.

Presence of increased CVD risk was categorized as 1) use of CVD medication such as statins or antihypertensives or 2) increased CVD risk evaluated by Systemic Coronary Risk Estimation 2 (SCORE2) algorithm for low-risk countries, including a 1.5 multiplication factor for all patients presenting with RA [2]. SCORE2 high risk thresholds of ≥ 2.5%, ≥ 5% and ≥ 7.5% were applied for ages < 50, 50–69 and ≥ 70 respectively [12].

Three questions pertaining to frequency, intensity and duration of habitual exercise were applied to generate a physical activity index as described by Nes et al. [36]. Additionally, exercise beliefs and self-efficacy were assessed by a composite score with unit weighing of twenty items over four domains; self-efficacy for exercise, barriers to exercise, benefits of exercise and impact of exercise on arthritis [37].

### Statistical analysis

Data are presented as mean ± standard deviation (SD) for normally distributed variables and median (IQR) for non-parametric variables. Categorical data are reported as counts and percentages. Normality was assessed by frequency histograms and quantile–quantile plots and in cases of doubt, a Shapiro–Wilk test was applied. Descriptive data includes complete case analysis, while single-case missing values were imputed by simple mean imputation for inferential analyses.

A multivariable linear regression analysis was applied to evaluate associates of CRF in our study population (VO_2peak_ in mL/kg/min as dependent variable). Based on clinical reasoning and literature review on correlates to CRF, the following variables were entered in our full model: age, gender, fat mass (in % of body weight), current use of cigarettes and/or snuff (yes/no), systolic blood pressure, HDL-c, physical activity index, disease activity and self-reported fatigue [35, 38–40]. With the significance level set to 0.05, the full model was reduced to the final model by backward elimination of non-significant variables. Gender and age were kept in the final model regardless of significance level. Model assumptions and model fit were evaluated by residual plots, variance inflation factors and Breusch-Pagan and Cook-Weisberg test for heteroskedasticity.

For each patient, measured VO_2peak_ was calculated in percent of Norwegian gender- and age-stratified reference data [35], and an 80% cut-off [41] was applied to dichotomize the study sample to *normal CRF*; VO_2peak_ ≥ 80% or *low CRF*; VO_2peak_ < 80% of reference values. Thereafter, we assessed potential between-group differences regarding anthropometric, CVD- and disease-related variables. Chi-square tests, or Fisher’s exact tests for expected values < 5, were applied for categorical data. Continuous data were analyzed by independent *t*-tests for normally distributed variables, Welch’s *t-*test if unequal variance and Wilcoxon rank-sum for non-parametric data. Due to multiple testing and the exploratory nature of these analyses, significance level was herein set to 0.01 with confidence intervals (CI) presented at the 99% level. All statistical analyses were performed using STATA version 16.1.

## Results

Demographic, cardiovascular and disease-related characteristics from baseline visits in the ExeHeart trial are presented in Table 1. In the sixty patients included in the present study (57% females), median age was 59 years (IQR 52–63). Mean VO_2peak_ in the study sample was 30.2 (± 6.9) mL/kg/min and median self-reported physical activity corresponded to the lowest value for the composite index (0, IQR 0–15). At the time of data collection, inflammatory markers CRP and ESR were low and disease activity was categorized to remission or low in 39 (65%) patients. Blood pressure and cholesterol levels were within normal range. Twelve (20%) patients reported current use of blood pressure medication and 34 (57%) patients used prescribed statins. Presence of increased CVD risk was observed in 49 (82%) patients.

**Table 1:** Descriptive statistics from baseline sessions in the ExeHeart study

Patient characteristics	*n* = 60
Age, years, median (IQR)	59 (52–63)
Gender, female, n (%)	34 (57)
Education > 12yrs, n (%)	46 (77)
Diagnosis	
Rheumatoid arthritis, n (%)	27 (45)
Spondyloarthritis, n (%)	19 (32)
Psoriatic arthritis, n (%)	14 (23)
Anthropometrics	
BMI, median (IQR)	25 (22.1–29.5)
Body fat mass, %, mean (SD)*	26.4 (7.3)
CardioPulmonary Exercise Test	
VO_2peak_ (mL/kg/min), mean (SD)	30.2 (6.9)
Respiratory exchange ratio, VCO_2_/VO_2_, mean (SD)	1.16 (0.07)
Borg RPE 0–10 (10 = maximal), median (IQR)	10 (9–10)
Percent of predicted peak heart rate (220-age), mean (SD)	101 (7)
Post-exercise blood lactate, mmol/L, mean (SD)§	9.4 (3.2)
IJD disease duration, years, median (IQR)	15.5 (7–30)
Inflammatory markers	
CRP, mg L^−1^, median (IQR)	1 (1–2)
ESR, mm h^−1^, median (IQR)	9 (5–15)
Disease activity categorized	
Remission, n (%)	22 (37)
Low, n (%)	17 (28)
Moderate, n (%)	14 (23)
High, n (%)	7 (12)
IJD medication	
Conventional DMARDS, n (%)	25 (42)
Biologics and/or JAK inhibitors, n (%)	43 (72)
Cortisone, n (%)	13 (22)
NSAIDs, n (%)	36 (60)
Analgesics	
Non-opioids, n (%)	42 (70)
Weak opioids, n (%)	7 (12)
Strong opioids, n (%)	0 (0)
CVD risk factors	
Systolic BP, mm Hg, mean (SD)	127 (13)
Diastolic BP, mm Hg, mean (SD)	83 (10)
Resting heart rate, beats/min, mean (SD)	68 (11)
Total cholesterol, mmol L^−1^, mean (SD)	4.8 (1.2)
HDL-c, mmol L^−1^, mean (SD)	1.7 (0.3)
LDL-c, mmol L^−1^, mean (SD)	2.6 (1.2)
Triglycerides, mmol L^−1^, mean (SD)	1.4 (0.7)
Current use of cigarettes/snuff, n (%)	13 (22)
SCORE2, %, median (IQR)^	4 (3–6)
CVD medication	
Statins, n (%)	34 (57)
Betablockers, n (%)	2 (3)
Blood pressure medication, n (%)	12 (20)
NRS (0–10), 0 = best	
Pain, median (IQR)	2 (1–4)
Fatigue, median (IQR)	3 (1–5)
Exercise beliefs and self-efficacy, (20–100, 100 = best), mean (SD)*	80.6 (9.0)
Physical activity index (0–45, 45 = best), median (IQR)	0 (0–15)

^§^*n* = 57 patients. *n = 59 patients. ^calculated in patients free from statins and blood pressure medication, *n* = 23 patients

*BP* blood pressure, *CRP* C-reactive protein, *CVD* cardiovascular disease, *DMARDs* disease-modifying anti-rheumatic drugs, *ESR* erythrocyte sedimentation rate, *HD-c* high-density lipoprotein cholesterol, *IJD* Inflammatory Joint Disease, *JAK* Janus Kinase inhibitors, *LDL-c* low-density lipoprotein cholesterol, *NRS* Numerical Rating Scale, *NSAIDs* non-steroidal anti-inflammatory drugs, *RPE* Rating of Perceived Exertion, *SCORE2* Systemic COronary Risk Estimation 2, *VCO*_*2*_ volume of carbon dioxide production, *VO*_*2*_ Volume of oxygen uptake, *VO*_*2peak*_ peak oxygen uptake

Table 2 outlines regression coefficients of VO_2peak_, significance level and 95% CI. In the full multiple regression model, age and fat mass (% of body weight) were significantly associated with VO_2peak_ (R^2^ = 0.68). Non-significant variables were dismissed in the following order; use of cigarettes/snuff, disease activity, HDL-c, systolic blood pressure and fatigue. In the final model, age and fat mass (% of body weight) were inversely associated with VO_2peak_, while an increase in physical activity index had a positive influence on VO_2peak_ (R^2^ = 0.66). There were no violations of regression assumptions.

**Table 2:** Variables associated with VO_2peak_ (mL/kg/min) assessed by multiple linear regression

Variable	Full model^a^, R^2^ = 0.68 Regression coefficients	95% CI	*p*-value	Final model^b^, R^2^ = 0.66 Regression coefficients	95% CI	*p*-value
Age, years	− 0.16	(− 0.31; − 0.01)	0.04	− 0.18	(− 0.31; − 0.04)	0.01
Female gender	− 1.56	(− 5.09; 1.97)	0.38	− 0.53	(− 3.09; 2.03)	0.68
Cigarettes/snuff, yes	0.24	(− 2.71; 3.19)	0.87			
Fatmass, % of body weight^	− 0.66	(− 0.88; − 0.43)	< 0.001	− 0.67	(− 0.85; − 0.48)	< 0.001
Systolic blood pressure, mmHg	0.04	(− 0.05; 0.13)	0.38			
HDL-c, mmol L^−1^	1.27	(− 1.89; 4.43)	0.42			
Physical activity index (0–45 points, 45 = best)	0.13	(− 0.01; 0.27)	0.08	0.13	(0.00; 0.27)	0.05
Disease activity category (1–4, 1 = remission)	− 0.49	(− 1.78; 0.79)	0.45			
NRS fatigue (0–10, 0 = best)	0.39	(− 0.19; 0.96)	0.18			
*Cons*	49.2			57.37		

**^**single missing data imputed by simple mean imputation

^a^Full model including age, gender, use of cigarettes/snuff, fatmass (% bodyweight), systolic blood pressure, HDL-c, physical activity index and NRS fatigue as independent variables

^b^Final model including age, gender, fatmass (% bodyweight) and physical activity index as independent variables

*HDL-c* High-density lipoprotein cholesterol, *NRS* Numerical Rating Scale

**Fig. 1:**
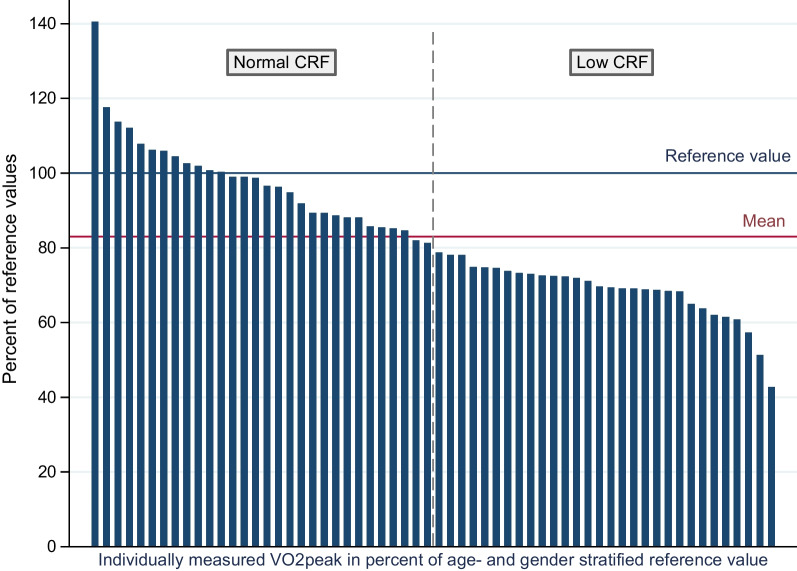
VO_2peak_ expressed in percent of Norwegian reference values. Blue line: Age- and gender-specific reference value from normative data [35]. Red line: Mean VO_2peak_ in percent of reference values. Dashed vertical line: Cut-off for normal versus low CRF. CRF: Cardiorespiratory fitness, VO_2peak_: Peak oxygen uptake

Mean VO_2peak_ corresponded to 83% (± 18) of normative data from the Norwegian general population (Fig. 1). Thirty patients (50%) presented with VO_2peak_ ≥ 80% reference values and were categorized to *normal CRF*, while the remaining 30 patients (50%) presented with VO_2peak_ < 80% and were classified as *low CRF* (Fig. 1)*.*

Significant differences were observed for the following variables in favor of patients with normal CRF: VO_2peak_, fat mass, resting heart rate, HDL-c, triglycerides and exercise self-efficacy. There were no significant differences regarding age, gender, education, diagnosis, disease duration, disease activity, blood pressure, total cholesterol, LDL-c, current use of cigarettes/snuff, presence of increased CVD risk, pain, fatigue, inflammatory markers, medication or physical activity index (Table 3).

**Table 3:** Exploratory between-group tests; normal CRF versus low CRF

Variable	Normal CRF (*n* = 30)	Low CRF (*n* = 30)	*p*-value	Estimated group difference (99% CI)
Age, years, median (IQR)	61 (55–63)	57.5 (50–62)	0.08^b^	
Gender, female, n (%)	17 (57)	17 (57)	1.0	na
Education > 12 years, n (%)	26 (87)	20 (67)	0.07	na
Diagnosis				
Rheumatoid arthritis, n (%)	12 (40)	15 (50)		
Spondyloarthritis, n (%)	12(40)	7 (23)		
Psoriatic arthritis, n (%)	6 (20)	8 (27)	0.38	na
Body fat mass, %, mean (SD)^	22.1 (5.2)	31.0 (6.6)	< 0.001^a^	− 8.7 (− 12.8; − 4.5)
VO_2peak_ (mL/kg/min)	34.9 (5.4)	25.6 (4.7)	< 0.001^a^	9.4 (5.9; 12.9)
IJD disease duration, years, median (IQR)	16.5 (10–30)	12.5 (7–23)	0.23^b^	
Inflammatory markers				
CRP, mg L^−1^, median (IQR)	1 (1–2)	2 (1–3)	0.05^b^	
ESR, mm h^−1^, median (IQR)	9 (3–16)	9 (5–15)	0.74^b^	
Disease activity categorized				
Remission, n (%)	11 (37)	11 (37)		
Low, n (%)	9 (30)	8 (27)		
Moderate, n (%)	9 (30)	5 (17)		
High, n (%)	1 (3)	6 (20)	0.20^c^	na
IJD medication				
Conventional DMARDS, n (%)	12 (40)	13 (43)	0.79	na
Biologics and/or JAK inhibitors, n (%)	20 (67)	23 (77)	0.39	na
Cortisone, n (%)	6 (21)	7 (23)	0.81	na
NSAIDs, n (%)	19 (63)	17 (57)	0.60	na
Analgesics				
Non-opioids, n (%)	20 (67)	22 (73)	0.57	na
Weak opioids, n (%)	3 (10)	4 (14)	0.71^c^	na
CVD risk factors				
Systolic BP, mm Hg, mean (SD)	127 (13)	127 (13)	0.94	− 0.3 (− 9.3; 8.8)
Diastolic BP, mm Hg, mean (SD)	83 (10)	84 (9)	0.46	− 1.8 (− 8.4; 4.7)
Resting heart rate, beats/min mean (SD)	64 (10)	72 (12)	< 0.01^a^	− 8.0 (− 15.4; − 0.7)
Total cholesterol, mmol L^−1^, mean (SD)	4.9 (1.3)	4.6 (1.2)	0.34	0.3 (− 0.5; 1.1)
HDL-c, mmol L^−1^, mean (SD)	1.9 (0.4)	1.4 (0.5)	< 0.001^a^	0.5 (0.1; 0.7)
LDL-c, mmol L^−1^, mean (SD)	2.6 (1.2)	2.6 (1.2)	0.93	0.0 (− 0.8; 0.8)
Triglycerides, mmol L^−1^, mean (SD)	1.1 (0.5)	1.6 (0.8)	< 0.01^a^	− 0.5 (− 1.0; − 0.1)
Current use of cigarettes/snuff, n (%)	4 (13)	9 (30)	0.12	n.a
Presence of increased CVD risk, n (%)	23 (77)	26 (87)	0.32	na
CVD medication				
Statins, n (%)	17 (57)	17 (57)	1.0	na
Blood pressure medication, n (%)	4 (13)	8 (27)	0.20	na
NRS 0–10, 0 = best				
Pain, median (IQR)	2 (1–4)	2 (1–4)	0.82^b^	
Fatigue, median (IQR)	3 (1–5)	3.5 (1–6)	0.52^b^	
Exercise beliefs and self-efficacy,				
(20–100, 100 = best), mean (SD)^	84.0 (7.3)	77.2 (9.4)	< 0.01^a^	6.9 (1.1; 12.7)
Physical activity index (0–45, 45 = best)				
median (IQR)	11.3 (0–15)	0 (0–0)	0.02^b^	

Comparison between groups by student *t*-test for continuous variables or chi-square test for categorical variables unless otherwise indicated.** ^**single missing data imputed by simple mean imputation

^a^Significant between-group differences

^b^Wilcoxon rank sum test

^c^Fisher’s exact test

*BP* blood pressure, *CRP* C-reactive protein, *CVD* cardiovascular disease, *DMARDs* disease-modifying anti-rheumatic drugs, *ESR* erythrocyte sedimentation rate, *HDL-c* High-density lipoprotein cholesterol, *IJD* Inflammatory Joint Disease, *JAK* Janus Kinase inhibitors, *LDL-c* low-density lipoprotein cholesterol, *NRS* Numerical Rating Scale, *NSAIDs* non-steroidal anti-inflammatory drugs, *SCORE2* Systemic COronary Risk Estimation 2, *VO*_*2peak*_ peak oxygen uptake

## Discussion

In the present cross-sectional study, age, fat mass and self-reported physical activity were associated with CRF (measured as VO_2peak_), and there were no significant associations between traditional CVD risk factors, disease activity and CRF in patients with IJD. Self-reported physical activity level was low, and in fifty percent of the patients, CRF was below 80% of reference values from the general population. Furthermore, in comparison to patients with CRF levels that aligned with normal reference values, patients with low CRF presented with higher fat mass, resting heart rate and triglycerides in concert with inferior HDL-c and exercise self-efficacy.

Our results complement extensive evidence that age and measures of anthropometry are known correlates of CRF in the general population [34, 38]. The non-significant association of gender and CRF in our data is puzzling, and we assume that the relatively small sample size may have obscured superior CRF values commonly observed in males [35, 42]. Our finding of a significant association of physical activity index to CRF parallels the work by Liff et al., where apart from BMI, level of physical activity was attributed the highest standardized coefficient of CRF in patients with RA [27]. Congruous data from an observational study in patients with RA confirmed inferior physical activity in patients at the lower end of the CRF spectrum [15], and in patients with SpA, physical activity measured by accelerometry was positively associated with CRF [14]. Conflicting results were reported from a recent case–control study involving patients with SpA, with no difference in physical activity despite higher estimated CRF in healthy participants [29]. Disparity in study results may be attributed to different population characteristics, methods of evaluating CRF and physical activity, and statistical modelling. Nonetheless, considering the well-known effect of physical activity on CRF, a connection between these two variables seems highly reasonable.

Discordant to our hypothesis, we unveiled no association between CVD risk factors and CRF in patients with IJD. Our results contrast prior observations of smoking status, blood pressure, serum lipids and resting heart rate as significant associates of CRF in other IJD samples [15, 26–28]. Four out of five patients in our cohort were categorized as having a CVD risk factor. Notably, measures of blood pressure and cholesterol were within recommended values [12]. Patients were recruited from a Preventive Cardio-Rheuma clinic and we assume that observed levels of blood pressure and cholesterol were duly influenced by adequate prescription of statins and/or antihypertensives. Blood pressure and cholesterol levels may relate to CRF in populations naïve to CVD medication, but our data indicate no such association in patients that are well managed in terms of these traditional CVD risk factors.

A plausible relationship between disease activity and CRF rests on assumptions that besides beneficial effects on CRF, exercise can upregulate anti-inflammatory cytokines and modulate the inflammatory profile in IJD [43, 44]. Contrary to our hypothesis, we detected no significant association between disease activity and CRF in our cohort. Paralleling our results, CRF was not correlated to composite measures of disease activity in Taiwanese [45] and Turkish [46] patients with SpA, and in UK patients with RA, estimated CRF did not correlate with disease-related variables [28]. However, other studies have reported an inverse association between ESR and CRF in patients with IJD [30, 47], and in the work by Metsios et al., both ESR and CRP were significantly elevated in RA patients categorized as unfit [15]. Interestingly, Liff et al. found that patient global assessment of disease activity was associated to CRF in patients with RA, but observed no relationship between clinical measures of disease activity and CRF [27]. This may serve to illustrate that despite the advent of biologic therapies and targets of disease remission in IJD, there may be discrepancies between what is captured by clinical measures of disease activity and the patient’s perception of disease burden. Considering the high prevalence of fatigue in IJD [48] and positive correlation to disease activity [49, 50], we addressed fatigue as a possible associate of CRF. Although few studies have assessed this relationship, low levels of physical activity correlate with fatigue in patients with IJD [40], and an inactive lifestyle can negatively impact CRF. There was no evidence of an association between fatigue and CRF in our data. However, median fatigue values were relatively low, and our unidimensional measure of fatigue may have fallen short of capturing various dimensions of fatigue in our patient cohort [51].

In the present study, CRF in patients with IJD was inferior to CRF in healthy Norwegian counterparts [35]. Observed values may differ from what is reported elsewhere, but direct comparison across studies is difficult due to variance in design, testing methodology and participant characteristics. Our finding of low CRF aligns with previous data of inferior CRF in Norwegian patients with RA and SpA [27, 47]. In support, Metsios et al. reported crucially low CRF in patients with RA [15], while O’Dwyer et al. observed inferior submaximal fitness in patients with SpA when compared to healthy peers [14]. Apart from interventional studies, there is currently a paucity of studies that have assessed CRF in patients with PsA.

Our exploratory analyses suggest that in IJD, patients with normal CRF present with favorable levels of fat mass, HDL-c and triglycerides in comparison to patients with low CRF irrespective of statin use. Although no causality can be inferred from these cross-sectional data, patients with normal CRF may have an increased exercise uptake and reap the benefits of exercise-induced changes in body composition and lipid profile [52, 53]. In alignment, Aspenes et al. observed advantageous cardiovascular risk profiles in individuals in the upper quartile of CRF, whereas an increased odds of CVD risk factor clustering was reported in the lowest CRF quartile [35].

In the context of IJD, motivation to exercise can enhance level of physical activity and CRF [54]. Our data signals lower exercise beliefs and self-efficacy in the least fit patients and supports the notion of superior CRF in patients that are motivated to exercise on a regular basis and believe exercise can benefit their IJD. Furthermore, we applied a physical activity index that assigns surplus weight to self-reported intensity of exercise. Although the significance level was just shy of our pre-defined alpha level, physical activity index was diminished in patients with low CRF, thus indicating that vigorous exercise is relatively uncommon in patients at the lower end of the fitness spectrum. These findings are not surprising, seeing as, aside from hereditary factors, vigorous exercise is a known determinant of CRF [4, 38].

Collectively, our results suggest that when disease activity and CVD risk factors are well managed in patients with IJD, associates of CRF parallel those of the general population. Future prospective studies are necessary to assess if fluctuations in CVD risk factors and disease activity can impact CRF in modern IJD care. Furthermore, repeated observations of reduced CRF implies presence of a CVD risk factor that needs to be addressed in order to optimize the cardiovascular profile and overall health in patients with IJD. Advocates of *‘Exercise is Medicine’* emphasize that pharmaceuticals have little influence on CRF, and call for integrated promotion and prescription of exercise as therapy [55, 56]. Our data supports a continued need to implement and evaluate exercise interventions to increase CRF and moderate CVD risk in patients with IJD.

### Strengths and limitations

A major strength of the current study is the use of the criterion method to quantify CRF [4]. Additionally, our choice of backward multiple regression with independent variables was driven by theoretical rationale as opposed to embracing significant variables from univariate analyses [57].

Limitations of the present study include absence of causal inference due to cross-sectional data. The data presented herein rests on baseline data from an RCT. We acknowledge the potential selection bias held by RCTs [58, 59] and that patients with low exercise self-efficacy may have been discouraged from participating. However, this may have introduced a positive bias to our CRF data given that patients disinterested in exercise trials are likely to present with even lower CRF than what was observed in our cohort. Patients were recruited from a Preventive Cardio-Rheuma clinic that emphasizes optimal management of CVD risk factors. Accordingly, CVD risk factors in our cohort may differ from other IJD populations. Our sample size is relatively small and although education level, smoking status and physical activity level are comparable to other Scandinavian IJD samples [13, 60], caution is advised in generalizing study results to the IJD population at large.

The sample size was leveraged by the power calculation for the ExeHeart RCT and associates of CRF may have been sequestered by low sample size (type 2 error). To avoid overfitting the regression model, we narrowed the IJD-specific independent variables in our analysis to include disease activity and fatigue. Instrument-specific thresholds were used to categorize disease activity across study participants with different IJD entities, and categorical data on disease activity may be too coarse to uncover a true relationship between disease activity and CRF. Furthermore, all our data on disease activity reflect current inflammatory burden and does not account for inflammatory burden over the course of disease.

A further limitation is the use of whole-body bioelectrical impedance analysis to quantify body composition. In bioelectrical impedance analysis, precision of fat mass and fat-free mass falls short of the reference method dual energy X-ray absorptiometry [61], and measures of body composition in the present study should be considered as approximates.

Lastly, we dichotomized CRF and recognize that even if creating binary variables is common in health research, cut-points are challenging to define and the practice of binary splits may be affiliated with loss of information [62]. Consequently, the results from the exploratory analyses regarding differences in patients with low versus normal CRF should be interpreted with caution.

## Conclusion

In the present study, age, fatmass and physical activity level were associated with CRF in patients with IJD. We observed lower levels of CRF compared to the general population, and our data suggests that patients with normal CRF may present with favorable levels of VO_2peak_, fat mass, resting heart rate, HDL-c, triglycerides and exercise self-efficacy. Our results support data headlining inferior CRF levels in patients with IJD, further illustrating a continued need for exercise interventions to improve CRF.

## Data Availability

The datasets used and/or analyzed during the current study are available from the corresponding author on reasonable request.

## Abbreviations

BMI: Body mass index
BP: Bloor pressure
CI: Confidence interval
CPET: Cardiopulmonary exercise test
CRF: Cardiorespiratory fitness
CRP: C-reactive protein
CVD: Cardiovascular disease
DMARDS: Disease-modifying anti-rheumatic drugs
ESR: Erythrocyte sedimentation rate
HDL-c: High-density lipoprotein cholesterol
IJD: Inflammatory joint disease
JAK: Janus Kinase inhibitors
LDL-c: Low-density lipoprotein cholesterol
PsA: Psoriatic arthritis
RA: Rheumatoid arthritis
RCT: Randomized controlled trial
SCORE2: Systemic coronary risk estimation 2
SD: Standard deviation
VCO_2_: Volume of carbon dioxide production
VO_2_: Volume of oxygen uptake
VO_2peak_: Peak oxygen uptake
